# Supplementary material for: Comparative Efficacy of Pyrethroid-Based Paints against Turkestan Cockroaches
Source: Insects. 2024 Mar 3;15(3):171. doi: 10.3390/insects15030171 (PMC10971201; doi:10.3390/insects15030171)
Supplement: Supplementary file 1 [file insects-15-00171-s001.zip › insects-2823269-SI.pdf]

Table S1. Mortality percentage (mean ± SE) at day 14 of cockroach nymphs exposed for 15 min or 1 h to fresh or 1-month aged insecticide-based paints on various substrates.

Insecticide	Treatment	Concrete	Metal	PVC
0.7% alphacypermethrin	Control	8 ± 6	6 ± 2	8 ± 6
	Fresh-15 min	12 ± 7	10 ± 4	10 ± 5
	Fresh-1h	34 ± 7	20 ± 5	20 ± 4
	1 month aged-1h	54 ± 17	82 ± 6	50 ± 10
0.25% deltamethrin	Control	8 ± 6	6 ± 2	8 ± 6
	Fresh-15 min	8 ± 6	10 ± 5	14 ± 4
	Fresh-1h	16 ± 5	10 ± 4	18 ± 9
	1 month aged-1h	40 ± 14	54 ± 9	44 ± 11
0.5% transfluthrin	Control	8 ± 6	6 ± 2	8 ± 6
	Fresh-15 min	8 ± 6	10 ± 6	6 ± 4
	Fresh-1h	14 ± 9	12 ± 8	14 ± 9
	1 month aged-1h	14 ± 2	12 ± 8	2 ± 2
